# Oxidative stress-induced by different stressors alters kidney tissue antioxidant markers and levels of creatinine and urea: the fate of renal membrane integrity

**DOI:** 10.1038/s41598-023-40454-5

**Published:** 2023-08-16

**Authors:** Bartholomew Chukwuebuka Nwogueze, Isioma Mary Ofili, Tochukwu Nnamdi Nnama, Chukwuemeka Peter Aloamaka

**Affiliations:** 1 Department of Physiology, Delta State University, Abraka, Delta State Nigeria; 2 Department of Nursing, Delta State University, Abraka, Delta State Nigeria; 3 Department of Anatomy, Alex Ekwueme Federal University Ndufu Aliko Ikwo, Abakaliki, Ebonyi State Nigeria

**Keywords:** Cell biology, Physiology

## Abstract

The cellular integrity of the kidney in homeostatic regulation has constantly been compromised by oxidative stress following exposure to varying nature of stressor present within the environment. The objective of the work was to evaluate the renal effect of the different stressor stimuli applied. Twenty-four adult female rats weighing averagely 160–200 g and within the ages of 12–14 weeks were used for experiment-1, while 12 offspring were utilized for experiment-2. Three stress models namely; restraint, mirror chamber and cat intruder stressors were used. Tissues were isolated from the animal and homogenized for tissue antioxidant assay. Serum was collected for assays of urea and creatinine for the kidney function test using ELISA. Data collected were analyzed for Mean ± SEM using One Way ANOVA. The present study revealed that exposure of rats to different stressors reduced relative kidney weights but did not significantly alter serum creatinine concentration in the Wistar rats, although the concentrations were slightly increased compared to controls. Urea concentration was significantly (*p* < 0.05) increased in rats exposed to restraint and intruder stressors. Exposure to a mirror chamber stressor did not significantly alter urea concentration. Offspring from parents of stressed female rats exhibited a significant (*p* < 0.05) increase in serum urea level, minimal increase in serum creatinine levels. GSH and GST levels showed no significant difference when compared to control group, whereas, GPx were significantly (*p* < 0.05) decreased irrespective of the stressor applied. SOD activity were significantly (*p* < 0.05) reduced in the group exposed to restraint or cat intruder stressor. CAT activities were significantly (*p* < 0.05) reduced in the rats exposed to restraint or cat intruder stressor. In all, the different stress model altered the antioxidant capacity of the kidney tissues. Exposure of rats to a stressful condition of the different nature of stressor has the tendency of compromising the functional integrity of the kidney, thus, with the potency of complicating female renal function.

## Introduction

Stress, is often characterized as an adaptive response which could either be physical, mental, or emotional, towards events capable of causing shifts in the homeostatic mechanism of an organism, allowing it to maximize its chances of survival when facing a stressor. Stress activates the hypothalamus pituitary–adrenal (HPA) axis, which then suppresses the hypothalamus–pituitary–ovarian axis^1,2^. This axis forms a complex set of interactions between the hypothalamus in the brain region, the pituitary gland, and the adrenal glands lying superior to the kidneys. The hypothalamus is the master control switch of the autonomic nervous system and a gland of the limbic system initiating both sympathetic and parasympathetic responses^3^.

Stress response is critical to the survival of an organism as is described by a period when the human body requires energy to protect itself against predatory or savaging creatures such as lion or saber-toothed tigers^4^. Maintaining an effective response to different kinds of stressors is physiologically useful and beneficial for survival^5^. Stress responses are connected with the set of physiological and emotional changes the body undergoes in response to a threat or stressor^6^. The effects of stress are often manifested in four varying folds such as, physiologic, subjective experience, behavioural, and cognitive functions depending on the individual’s predictability and controllability^7^. Increase in stress level has been linked to excessive reactive oxygen species production, leading to impaired kidney integrity and cellular membrane function^8,9^.

Oxidative stress connotes an imbalance which occurs in an organism between the oxidative free radicals produced following stressful conditions and the levels of antioxidative systems protecting the cellular architecture, such imbalance according to Sarandol et al*.*^10^, occurs when it is in favour of oxidative radicals. Ishii et al*.*^11^ affirmed that oxidative stress is a state of increased cellular damage occurring when there is excessive production of reactive oxygen species (ROS) than the body’s antioxidant defenses^12^. Antioxidants act by neutralizing free radicals in humans, rodents and other species^13^. In the stressful situation in which the rats found themselves, the various organs in the rats were faced with relatively high production of ROS^14^, comprising their respective structural and functional integrity^13,15,16^, such as the organ of the kidney. Hence, this study examined the effect of oxidative stress on renal antioxidant integrity (GSH, GS, GPx, SOD, CAT and MDA), Creatinine, and Urea Levels in Wistar rats.

## Materials and methods

### Research design

Twenty-four (24) healthy adult female Wistar rats aged between 12 and 14 weeks and weighing between 160 and 200 g were used for experiment 1, whereas, for the second experiment using offspring, 12 rats from stressed parents were used adopting the technique described by Nwogueze et al*.*^17^ the rats in this study were stressed for 3 h per day for a period of 3 weeks. The study was carried out in Physiology Department, Faculty of Basic Medical Sciences, Delta State University, Abraka, Nigeria. The study covered a period of 14 weeks.

### Animal grouping and handling

The experimental rats used in the study were purchased from the Animal House of the Department of Anatomy, Delta State University, Abraka. The rats were randomly assigned into 4 groups of six (6) rats. The Group 1 served as the control group that were not stressed. The Group 2 were rats exposed to restraint stress model, group 3 were rats exposed to mirror chamber stress chamber and group 4 were rats exposed to cat resident intruders. The experimental rats were allowed to acclimatize for 2 weeks and were fed with standard rat chow and water ad libitum. The rats were housed in wire mesh cages under clean environment and good environmental conditions of temperature ranging between 20 and 26 °C with 30–70% humidity, in addition to normal 12 h day and 12 h dark cycle.

### Stress induction

The rats were induced using three different nature of stressors such as, stress model to induce physical stress, mirror chamber stressor to induce anxiety, and cat resident intruder stressor to induce psychosocial stress as demonstrated by Nwogueze et al*.*^17^.

### Biochemical analysis

After the completion of the stress induction period of 3 weeks, the rats were euthanasia by cervical dislocation and blood samples were collected for biochemical investigations for urea and creatinine following the methods described below. Kidney tissues were also carefully harvested and homogenized for activities of Glutathione antioxidant (GSH, GST and GPx), antioxidant enzymes (SOD and CAT) and MDA levels adopting the methods described by Nwogueze et al*.*^12^.

### Serum creatinine assay

Creatinine in alkaline solution reacts with picric acid to form a colored complex. The amount of the complex formed is directly proportional to the creatinine concentration. Randox test kit was used for this estimation. Using fresh double-distilled water (ddH_2_O), performed a new Gain Calibration in cuvette mode. Select CREA in the Run Test screen and carry out with water blank as instructed in the test manual. The reaction rate and absorbance of the reaction product are very sensitive to temperature. The specified temperature must therefore be maintained.

### Serum urea assay

Urea is hydrolyzed in the presence of water and urease to produce ammonia and carbon dioxide. The ammonia produced in the first reaction combines with α-oxoglutarate and NADH in the presence of glutamate dehydrogenase. Using fresh ddH_2_O, performed a new Gain Calibration in flow cell mode. Select UREA in the Run Test screen and carry out a water blank as instructed. Randox test kit was used for this estimation.

### Data analysis

The data were analyzed for Mean ± SEM using the Statistical Package for Social Sciences (SPSS), version 20. One Way ANOVA was used for multiple comparison across of non-parametric variables. Fisher’s Least Square Difference was employed to test for post hoc association between variables. Significance is measured at *p* < 0.05.

### Ethical approval

All experimental protocols were approved by the Research, Bio-ethics and Grants Committee on animal experiments of the Faculty of Basic Medical Sciences, Delta State University, Abraka with approval No: REC/FBMS/DELSU/19/58. All methods were performed in accordance with ARRIVE guidelines 2.0 and experimental regulations on animal care and monitoring.

## Results

**Figure 1:**
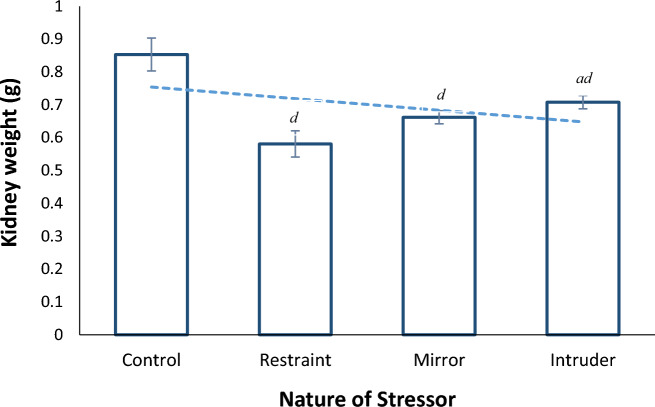
Kidney organ weights of Wistar rats Exposed to different stressors for 3 h per day for 3 weeks. Data obtained is expressed as mean and standard error bars. ^a^significant when compared to the restraint stressor. ^d^significant when compared to the control group.

Figure 1 shows the kidney organ weights of female non-pregnant Wistar rats exposed to different stressors (Restraint, mirror and intruder) at the rate of 3 h per day for 3 weeks. The kidney of the rats exposed to the three stressors significantly (*p* < 0.05) decreased the weight of the organs when compared to their control weights. The kidney weight of rats exposed to the restraint stress model was significantly (*p* < 0.05) decreased when compared to those of rats stressed by exposure to the cat intruder stressor.

**Figure 2:**
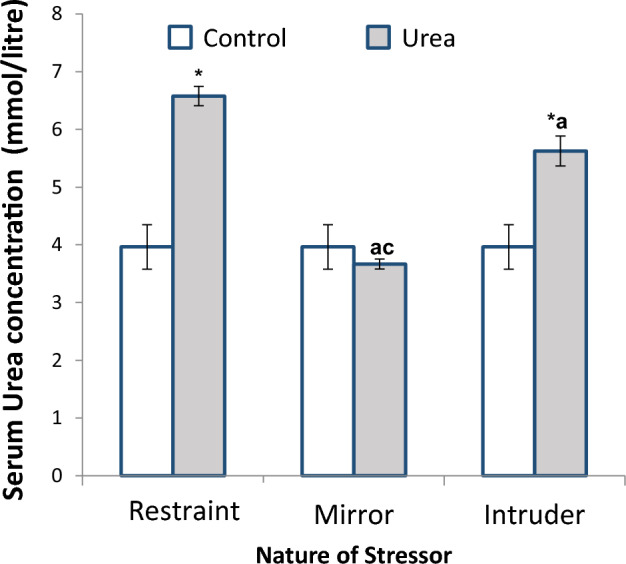
Effect of stress on urea concentration of female non-pregnant wistar rats after exposure to restraint, mirror and intruder stressors for 3 weeks at the rate of 3 h per day (n = 6). Data obtained is expressed as mean and standard error bars. *significant when compared to the control group. ^a^significant when compared to the restraint stressor. ^c^significant when compared to the cat intruder stressor.

Figure 2 shows the comparative effect of stress on serum urea concentration of female non-pregnant Wistar rats after exposure to restraint, mirror chamber and cat intruder stressors respectively at the rate of 3 h per day for 3 weeks. There was a significant increase in urea concentration in rats exposed to restraint and cat intruder stressors when compared to the control group (*p* < 0.05). The urea level in rats exposed to the mirror chamber stressor was not significantly altered, but was significantly (*p* < 0.05) less than the rats stressed by exposure to restraint and cat intruder stressors.

**Figure 3:**
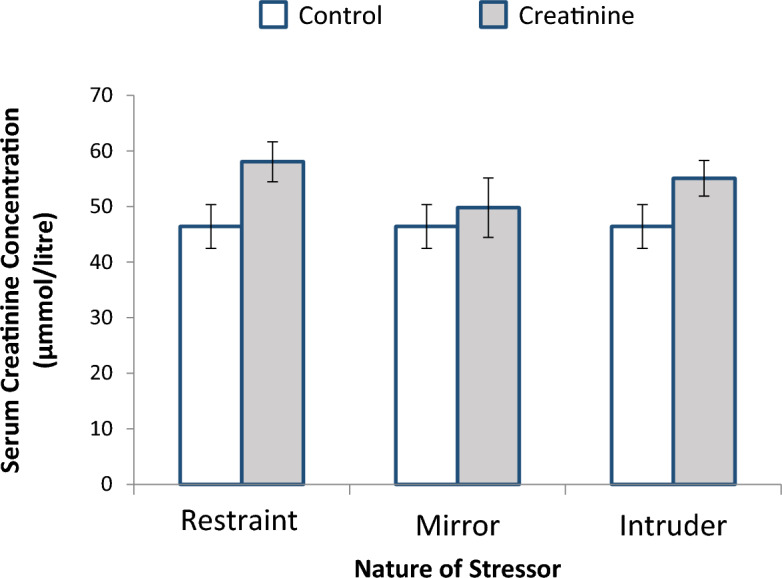
Effect of stress on creatinine concentration of female non-pregnant Wistar rats after exposure to restraint, mirror and intruder stressors for 3 weeks at the rate of 3 h per day (n = 6). Data obtained is expressed as mean and standard error bars.

Figure 3 shows the comparative effect of stress on serum creatinine concentration of female non-pregnant Wistar rats after exposure to restraint, mirror chamber and cat intruder stressors at the rate of 3 h per day for 3 weeks. Irrespective of the nature of the stressor to which the rats were exposed to, exposure to stress elevated the serum concentrations of creatinine, although the increase was not significant.

**Figure 4:**
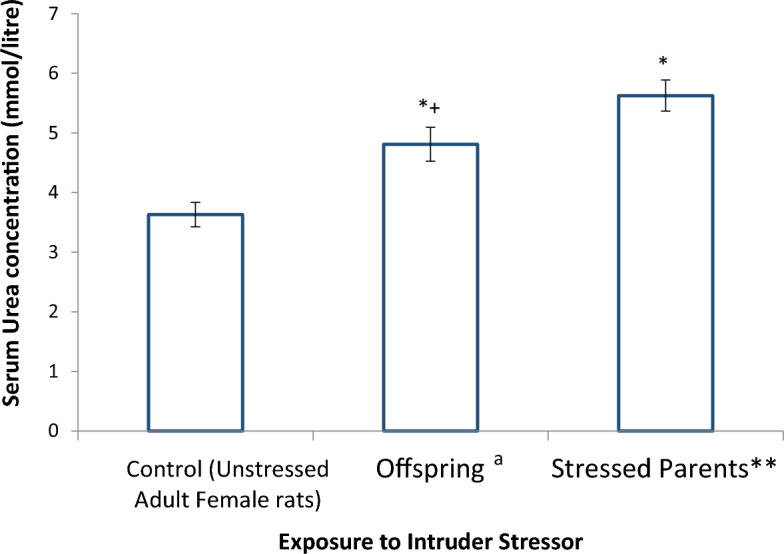
Effect of stress on serum urea concentration of stressed Wistar rats parents and their female offspring (n = 6). *significant (*p* < 0.05) when compared to the control group. +significant (*p* < 0.05) when compared to the stressed parent rats. **stressed by exposure to cat intruder stressor. ^a^blood was collected at 4 weeks after delivery.

Figure 4 shows the comparative effect of stress on serum urea concentration of stressed parents (female Wistar rats) and their unstressed female offspring. The stressed parent rats were stressed with the cat intruder stressor by exposing the rats to the stress for 3 weeks and at a rate of 3 h per day. The serum urea concentration of the offspring of the stressed parents was significantly (*p* < 0.05) different from that of their parents, and both urea concentrations were significantly (*p* < 0.05) greater than that of the control rats.

**Figure 5:**
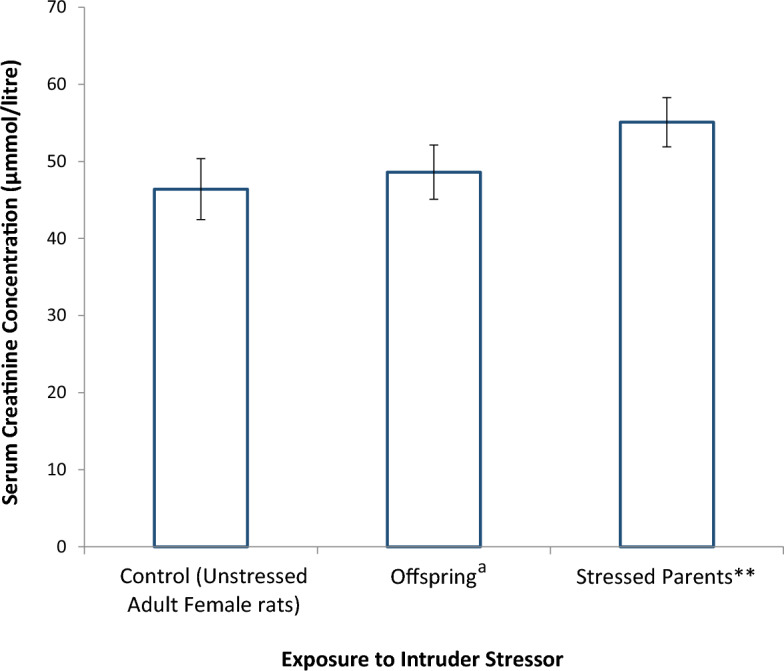
Effect of Stress on Serum Creatinine Concentration of Stressed Parent Wistar Rats and their Female Offspring (n = 6). **stressed by exposure to cat intruder stressor. ^a^blood was collected at 4 weeks after delivery.

Figure 5 shows the comparative effect of stress on creatinine concentration of stressed Wistar rats, stressed parent rats, and their unstressed female offspring. The parent rats were stressed with the cat intruder stressor by exposing them to the stress for 3 weeks and at the rate of 3 h per day. Serum creatinine concentration of both the parent rats and their offspring increased, but the increase was not significant when compared with the serum creatinine level of the control rats. The serum creatinine concentrations of the offspring and their parents were not significantly different.

Table 1 represents the results of experiments for assessment of the levels of glutathione oxidative stress activity of the kidney tissues of stressed female Wistar rats following exposure to nature of stressors at the rate of 3 h per day for 3 weeks. The kidney GSH levels and GST activity of rats exposed to restraint or cat intruder stressor were not significantly different from the levels in the control group. With respect to rats stressed by exposure to the mirror chamber stressor, there was a statistically significant (*p* < 0.05) increase in the level of kidney GSH and GST activities when compared to the control levels. Exposure of the rats to any of the stressors did not significantly alter the activity of kidney GPx when compared with GST activity of the control rats, despite the observed reduction. However, kidney GPx activity was significant (*p* < 0.05) reduced when comparing kidney GPx of rats exposed to mirror chamber and restraint stressors to those exposed to the cat intruder stressor.

**Table 1:** Stress-induced modulation in glutathione levels in kidney of female Wistar rats exposed to restraint, mirror or intruder stressor (n = 6).

Stressors	Duration of exposure to stress (week)	Stress exposure rate (hours per day)	Kidney glutathione antioxidant activity		
			GSH (µM/mg protein)	GST (µM/mg protein)	GPx (µM/mg protein)
Control	–	–	43.24 ± 1.08	38.50 ± 1.12	39.68 ± 1.69
Restraint	3	3	42.14 ± 1.99	37.36 ± 2.06	30.50 ± 1.56^d^
Mirror	3	3	46.75 ± 1.13^a^	42.12 ± 1.17^a^	29.05 ± 0.88^d^
Intruder [[	3	3	43.73 ± 1.39	39.01 ± 1.43	30.06 ± 1.17^d^

^a^Significant (*p* < 0.05) when compared to Restraint stressor.

^d^Significant (*p* < 0.05) when compared to Control group.

Table 2 shows stress-induced Superoxide Dismutase (SOD), Catalase (CAT) antioxidant activity and Malondialdehyde (MDA) levels of the kidney tissues of stressed female Wistar rats exposed to different nature of stressors at the rate of 3 h per day for 3 weeks. The activity of kidney SOD of rats stressed by exposure to restraint, mirror chamber or cat intruder stressors was not altered significantly when compared to the activity of the enzyme in the control rats. In the case of kidney tissues of rats stressed by exposure to restraint or cat intruder stressors, the activity of SOD decreased significantly (*p* < 0.05), when compared to the control SOD activity. But when the rats were exposed to mirror chamber stressor SOD activity significantly (*p* < 0.05) increased. Exposure to restraint or mirror chamber stressors significantly (*p* < 0.05) decreased the activity of CAT in the organ. But exposure to intruder stressor had no significant effect on the activity of CAT in the kidney tissues of the stressed rats, as the activity was not significantly different from that of the control groups. Stressing of the female rats by applying the cat intruder stressor significantly (*p* < 0.05) increased the level of MDA in kidney of the stressed rats beyond the level in the respective organs of the control rats. But when the stressor was of the restraint or mirror type, MDA level was not significantly altered when compared with the levels in the control rats.

**Table 2:** Stress-induced SOD, CAT antioxidant enzymes and MDA levels in kidney of female Wistar rats Stressed by exposure to restraint, mirror or intruder stressor (n = 6).

Stressors	Duration of exposure to stress (week)	Stress exposure rate (hours per day)	Antioxidant enzymes		
			SOD (U/ml)	CAT (U/ml)	MDA (nmol/ml)
Control	–	–	29.60 ± 1.51	22.48 ± 0.50	0.22 ± 0.03
Restraint	3	3	22.00 ± 2.70^bd^	18.54 ± 0.65^ cd^	0.37 ± 0.08
Mirror	3	3	36.89 ± 1.60^d^	14.70 ± 0.07^ cd^	0.27 ± 0.04
Intruder [[	3	3	18.93 ± 1.89^b^	21.33 ± 0.95	0.42 ± 0.07^d^

^b^Significant (*p* < 0.05) when compared to Mirror chamber stressor.

^c^Significant (*p* < 0.05) when compared to Intruder group.

^d^Significant (*p* < 0.05) when compared to Control group.

Table 3 shows the antioxidant profiles in the kidney of rats stressed by exposure to different nature of stressors at the rate of 3 h per day for 3 weeks. The three stressors involved were potent stressors. The restraint stressor caused a significant (*p* < 0.05) decrease when compared to the control group for the GPx, SOD, and CAT activities. It must be noted that the impact was more with respect to rats exposed to mirror chamber stressor, the kidney GPx, and CAT were significantly (*p* < 0.05) decreased when compared to the control group, whereas, SOD activity was significant (*p* < 0.05) increased when compared to the control group. Similar remarks can be made for the cat intruder stressor, the kidney GPx and SOD were significantly (*p* < 0.05) decreased when compared to the control group, except that the kidney MDA level was significantly (*p* < 0.05) increased.

**Table 3:** Antioxidant profiles in kidney of rats stressed by exposure to different stressors for 3 weeks at the rate of 3 h per day.

Antioxidants	Nature of stressors		
	Restraint	Mirror	Intruder
GSH	n	N	n
GST	n	N	n
GPx	↓^*ed*^	↓^*ed*^	↓^*ed*^
SOD	↓^*ed*^	↑^*ed*^	↓^*ed*^
CAT	↓^*ed*^	↓^*ed*^	n
MDA	n	N	↑^*ed*^

↑^*ed*^ Significant (*p* < 0.05) increased when compared to the Control group.

↓^*ed*^ Significant (*p* < 0.05) decreased when compared to the Control group.

n No Significant change.

## Discussion

The different internal organs of animals perform different functions and this is so because, apart from structural differences, the chemical (enzymatic and non-enzymatic) constituents of the different organs vary, thereby giving rise to different chemical reactions in the cells of the affected organs. Exposure to the three different nature of stressors significantly (*p* < 0.05) decreased the weight of the kidney of the rats. This agrees with the findings reported by Nayanatara et al.^18^ that reported variable effect of exposure of rats to stressors on different organ weights. A previous work by Nwogueze et al.^19^ confirmed that weights of the brain and kidneys were significantly (*p* < 0.05) decreased following exposure to restraint, mirror or resident intruder stressors depending on time and duration. Inversely, Liu et al*.*^20^ found out that exposure to restraint stress increased kidney, liver, and brain weights of rats. However, based on available data from this study, it is not possible to provide precise mechanisms for the variable responses of renal kidney weight following exposure of rats to a particular stressor.

The results from the study have also shown that the functional integrity of the kidneys of rats exposed to stressful conditions is compromised. Serum urea concentration was significantly increased in rats exposed to the restraint and cat intruder stressors, but this was not the situation when the rats were exposed to the mirror chamber stressor. In the latter case, serum urea concentration was not significantly altered. Urea is a final metabolite of protein nitrogen balance and it is excreted from blood primarily by the kidney via glomerular filtration. Serum concentration of urea beyond its normal physiological range will suggest abnormality of tubular glomerular filtration^21^. Although, the estimation of serum plasma concentration of urea is still in use in clinical examination of the functional integrity of kidneys, it is usually conducted along with the estimation of the serum level of creatinine. According Borges et al*.*^22^ urea concentration is the first acute renal marker. When increased it indicates dysfunction and injury to the kidney; otherwise, an increase in plasma creatinine level is the most trustable renal marker, which occurs when the majority of renal function is lost.

Creatinine is an excretion product of muscle activities circulating in the blood, and its elimination is exclusively through the kidney^23^, unlike urea which can also be eliminated via other routes such as in sweat. It was observed that exposure to the three stressors studied did not significantly alter serum creatinine concentrations in the Wistar rats, however the concentration in each case was slightly increased when compared to control levels. Although, exposure of the rats for 3 weeks to the three different nature of stressors did not significantly alter serum creatinine concentration, it is possible that if the exposure continues for much longer period, the concentration will further increase—suggesting that the three stressors have the potential for causing renal damage. This fear is not misplaced as pointed out in previous studies^24,25^, however, with respect to other stressors, they observed deleterious changes in renal function following exposure to stressful conditions. More so, in our study, the kidney functions of offspring of stressed rats (cat intruder stressor) were adversely affected. Offspring of stressed female Wistar rat parents exhibited a significant increase in serum urea levels, minimal increase in serum creatinine levels.

In the present study, the kidney GSH levels of rats exposed to restraint and intruder stressors were not significantly different from the levels in the control group. The level of GSH in the kidney of exposed rats exposed to mirror chamber stressor was higher than the control rats. Mehri et al*.*^26^ found decreased GSH levels in kidney and brain tissues from acrylamide stress-induced toxicity and according to Park et al*.*^27^ severe decrease in liver GSH content leads to degeneration and necrosis of hepatic paramchyma in stressed rats. In all, if the antioxidant capacity in the respective tissues is not enhanced by an increase in the production of some specific antioxidants, the tissues concerned will be prone to tissue injury^28^. Thus, a decrease of reduced GSH in these kidney tissues results overly in lower rates of peroxidase activity, alteration of the integrity of the kidney membrane, and hepatotoxicity^29^.

Glutathione-S- Transferase (GST) represent a complex family of proteins that catalyze the deactivation of harmful compounds by electrophilic centers towards ensuring their excretion from the cell^30^. Our results revealed that the kidney GST activities of rats exposed to restraint or cat intruder stressor were not significantly different from the levels in the control groups. However, exposure to mirror chamber stressor caused elevation in the level of kidney GST activity when compared to the control levels. The different observations made in their study could be because of the nature of the stressors used and the duration of exposure of the rats to that stressor, considering that the rats were under oxidative stress, perhaps, Nwogueze et al*.*^29^ suggested in an earlier study that the anti-oxidant capacity in the respective organs exposed to resident intruder, mirror and restraint stress models having variable effects has been compromised, thus there is inadequate levels of GST in scavenging free radicals that are generated by the oxidative stress. Glutathione peroxidase is an antioxidant found in the cytosol and mitochondria of cells, acting as a detoxifying agent by participating in the GSH redox cycle^31^. Exposure of the rats to any of the stressors caused a reduction in the activity of kidney GPx, although not significant when compared with the GST activity of the control rats. According to Chatziargyriou and Dailianis^32^, GPx is one of the key enzymes vital in regulating glutathione homeostasis and sustaining cellular integrity in tissues. Thus, in our study, the activity of GPx in the kidney of rats stressed by exposure to the different nature of stressors applied, was either decreased or unaltered from the control level.

Results from this study showed that the activity of kidney SOD of rats following exposure to restraint and cat intruder stressors were significantly reduced, indicating an unsafe pathogenic lesion to the kidney tissues in stressful conditions. But the activity was increased in the kidneys from rats stressed by exposure to the mirror chamber stressor. Similar study by Ohta and Nishida^33^, reported that SOD activity was increased in stressed rats, however, the changes was reported in the stomach tissues. Stressing the rats by exposure to restraint or mirror chamber stressor significantly (*p* < 0.05) decreased the activity of CAT in the kidney. Similar effect relating to the reducing effect on CAT caused by stress induction using restraint stressor on the kidney tissue has been reported in previous study by Ayesha and Naheed^34^, as well as study by Iman and Yasser^35^, who induced stress using bisphenol. The mechanism of stress-induced MDA in kidney tissues involves the interactions between cell proteins and lipids resulting in the generation and release of free radicals, and consequently cellular damage, which at an extreme level interferes with the structural and functional integrity of cells and their respective organelles’ membrane^36^.

## Conclusions

In conclusion, our findings revealed that kidney weights of the stressed Wistar rats were markedly reduced following exposure to the different stress models. Serum creatinine levels were unaffected by application of the different stressors, although the concentrations were slightly increased compared to control. Urea concentration was significantly increased in rats exposed to restraint or cat intruder stressor, whereas exposure to mirror chamber stressor did not alter urea concentration. From our results it was obvious that exposure of the rats to various stressors compromised the antioxidant defense capacity of the organ, with no exception. In all, continued exposure of rats to stressful conditions has the tendency of compromising the functional integrity of the kidney and antioxidant capacity of the kidney against oxidative stress. It is recommended that a histopathological investigation should be conducted in this regard as this will contribute largely to future clinical medicine.

## Data Availability

Data used in the study will be made available on request from the corresponding author.
